# Pathology and Therapeutics—From a Conservative "New Departure" Standpoint

**Published:** 1881-02

**Authors:** F. E. Howard

**Affiliations:** Geneseo, N. Y.


### ARTICLE IV.

### *Pathology and Therapeutics—from a Conservative "New Departure" Standpoint.*

BY F. E. HOWARD, M. D. S., GENESEO, N. Y.

(Read before the 7th and 8th District Dental Societies, October 26, 1880, at Rochester, N. Y.)

Pathology, respecting secondary decay, is viewed very differently by many at the present day, from what it was five years ago. Up to about 1875, it was generally supposed that secondary decay about fillings arose from "exactly" the same causes that the original disease was produced, and is identically the same thing,—"via" "decomposition of tooth structure, the result of solvent action of acids, which have been generated by fermentation going on in the mouth;" or the "chemical union of mineral constitutents of the tooth, with elements contained in the fluids to which it is exposed;" the result of wanton carelessness, or inability on the part of the patient to maintain absolute cleanness about fillings.

When secondary decay did occur about gold fillings, and it could not be charged to the results just mentioned, it was attributed to "defective manipulation." Operations were repeated from time to time, and repairs made with the same results, by "this faction" of the profession.

Another class of the profession were not satisfied with this explanation of the cause of secondary decay about fillings, and proceeded to investigate the subject, from a *scientific standpoint.* The result of such investigation has led to the development of new theories, termed the "new departure." They maintain that secondary decay about fillings may be, in many cases, the result of incompatibility between filling and tooth bone. The result of conductivity of fillings. And that the successful treatment of many cases require a physiological change in the organ, or therapeutic and antiseptic treatment, not "recognized" prior to these investigations. They claim that at certain stages of decay, a metallic filling (we will take "gold," as this is the best conductor,) promotes decay, and in many cases it is the *prime agent in provoking it.* This is the view we are compelled to accept, from a careful, practical observation, in every day practice.

When this is manifest, it is shown in that class of poorly calcified teeth, and in those of young subjects, where the "aqueous" or vital element is in excess of the mineral constituent. If these teeth are filled with gutta-percha or any non-conducting material, though the fillings may soon be worn away by attrition, the decay is absolutely stopped so long as the material remains in a position to protect the walls of the cavity from external causes of decay; such as preventing the ingress of particles of food, and foreign substances in general, even though "dampness" pervade the whole *cavity underneath the filling.* And there are few cases where gutta-percha fillings are water tight. In this class of fillings though often imperfectly done, decay in the very poorest quality of teeth is effectually arrested.

Why so? "Because the aqueous element of the tooth, being of the same nature as the solid constituent, has no power to act upon the latter, and cannot cause decay, or dissolution, until the equilibruim is disturbed." "Decay only takes place, when the external fluid is rendered chemically different by confinement in fissures, pits, or cavities, and is thus changed from the other fluids, to which other surfaces of the teeth are exposed." The non-conducting filling being in harmony with the tooth, the aqueous element remains neutral or becomes neutral by contact with this vital fluid. This is the case with leaky non-conducting fillings, consequently there is nothing to excite chemical action. The decay is arrested by external excitants being shut out, such as thermal changes, electrical currents, and decomposing fluids, and nature goes on unmolested in the work of more perfect calcification of the organ.

This, together with the usual antiseptic and disinfectant remedies, is the topical, therapeutic treatment that such cases require. And yet, this is not the course that is best to pursue in all cases. It is often advisable to use a material that is antagonistic and incompatible with the tooth structure. Under these circumstances we must anticipate future decay, and make calculations accordingly with the idea of refilling, or repairing, at no distant future. Because if we fill these poor, soft, or imperfectly calcified teeth with gold, we must depend upon it, the aqueous element, natural to this class of teeth, is in a condition that is liable under many circumstances to work mischief, despite all efforts on our part, not excepting those of even a few of the best operators we have.

In this we mean to show that we have an element to contend with, that mere mechanical means will not overcome. Gold is not a "panacea" for all conditions of decay of the teeth, any more than some "patent medicines" are for all ills that flesh is heir to. For an example, we will take gold, and fill this class of teeth in such localities as proximate, and labial surfaces, and the filling will often exert a deleterious condition. There is a certain class of teeth that we have to operate on, that we find affected by decay—sometimes it might be termed erosion more properly—where the lime salts are dissolved out at a line ascribed by the gum, white opaque patches are presented that can often be removed with the thumb nail, being so soft, and chalky, caused by the dissolution of earthy matter occasioned by acid secretions. Now we must admit, that if we prepare a cavity of this sort, and fill with gold, the margins about the fillings seem to fairly melt away, like snow beneath the noon-day's sun. This is not always the result of imperfect manipulation; if these fillings are absolutely water tight in themselves, the result may be the same. Often there is so much of the aqueous element in the organ, that it is impossible to dispose of moisture about the fillings for any great length of time, no matter how skillfully the operation may have been performed. For in this class of teeth there seems to be, under some circumstances, a *retrograde action*, that "supercedes" the topical therapeutic treatment, that may have been applied to the cavity before filling.

And this is one thing that the opponents of this theory have lost sight of, or would not look at squarely in the face, but hang their faith on this peg; that if there is no leakage about a gold filling there can be no electrical action; and if our fillings are water-tight, so as to perfectly exclude external moisture, we have accomplished all that is necessary for success. A few have recognized this in a measure, but claim where this aqueous condition is manifest, it is confined to the dentinal tubuli, and not so with the enamel—the latter being of such a dense structure is devoid of this aqueous element entirely. And that if a filling at the margins be perfectly water-tight, the moisture from the dentinal tubuli will be inert to act deleteriously upon the tooth—for no electric current can be established.

But this is an error, and not the case at all. For it has been shown conclusively by Dr. Abbott, (and I think others,) that "the enamel is not *a crystal but a living tissue*, endowed with the same vital element as dentine, only to a less degree." Now that this is an established fact, it is our conviction that a filling often becomes a battery, in many cases, where the operation is "absolutely perfect." And the result is electro-chemical decomposition about the margins of fillings, in spite of our best efforts.

Doubtless many of you have seen discoloration about fillings made with "crystal gold" some years ago, of a bluish character, that is generally pronounced "leakage." In fact this gold was discarded by many, because of the discoloration that often followed its use. In many cases, this was not the result of faulty operations. I have seen many of these fillings, inserted by eastern gentlemen of acknowledged ability, whose operations with gold are perfection. About many of these fillings there was a "blueness" which was proof positive of chemical action. The acid used in the preparation of this gold, had left its mark. The iron had entered the tubuli, and the aqueous element in the tooth was fuel sufficient to feed this chemical fire. In such conditions, with soft teeth time generally pronounces the verdict of failure. With well calcified ones, the power of resistance to such impressions may prove a success.

When we think of the recognized make up of the different classes of teeth and hear the claim made, that gold is the best filling for all teeth, or as good as any, if properly manipulated, it reminds me of something that has been said by Dr. S. B. Palmer on this subject, and it goes to show how absurd it would be to apply this principle of gold in all cases, to other matters outside of the mouth, where judgment should govern our operations. He says: "In case of repairing a leaky cistern, wisdom would demand knowledge of the material of which the cistern is composed, and the nature of the repair necessary. Then call a carpenter, plumber, or mason to make the repair. A porous cement cistern would not be benefited by the carpenter or plumber, only by a mason, and the application of a chemical to fill the pores over the entire surface." * * *

And in this class of teeth mere mechanical manipulation of a material that is indestructible in itself, cannot be made subservient to meet the requirements of all cases.

Dr. Flagg uses no gold in his own practice at the present day. Yet this is not the teaching of the new theory. He says, "that the 'New Departure' concedes the use of gold as a filling material whenever! and wherever!! it can be used for the preservation of teeth as successfully as other materials. The new departure begins where gold leaves off, to help gold if need be, to sustain its exalted reputation, and to furnish a substitute in other materials, in cases where gold is inadmissible."

And he remarked at the outset in presenting his paper to the Odontological Society, New York, in 1877, which excited so much discussion and comment: "I do not wan to say anything to you of the teeth which you are in the habit of filling successfully, and as we express it, satisfactory with gold—teeth of dense structure, whose cavities have walls so strong that you can impact a filling which lasts a life-time. But I do ask, that you will gradually discontinue this packing of gold into teeth that are so poor, so frail, so unsubstantial, that it is, to say the least, doubtful whether the result will be creditable to your profession, or satisfactory to your patient."

This is the point I wish to make. And it must be admitted by any intelligent, unbiased dentist, that many of the non-conducting fillings that we have at our command, meet the requirements of such teeth better though the filling may be short-lived in itself. But it is really the topical therapeutic treatment, that such cases often demand

Who is there, that has practiced for some years, that has not groups of gold fillings on labial surfaces, one adjoining another, put in at different times; or removed and renewed the whole operation, from time to time? We do not denounce the practice; often we are compelled to choose the least of two evils. If the subject cannot have the case renewed from time to time, when the plastics are used up, (for in such localities, gutta-percha, oxychlorides, and the like, are soon worn down by friction and attrition. I trust we have something better in the "Oxy-phosphate." The latter I have experimented with for two (2) years very satisfactorily. But this is scarcely time enough to determine its durability, and in what class of mouths, and what condition of the secretion, they will do the best service. And when this class of plastic fillings are not admissible in our judgment, according to the varied circumstances that may be presented,) we must do the next best thing, which would be to devote much more time in the operation, and increase the expense of a gold filling, often at the expense of the vital organization of the tooth, instead of having that soothing antiseptic and therapeutic condition imparted to the organ, by the non-conducting fillings. The latter assists nature in the work of perfect development; while the former "somtimes" retards this progress, for under chemical excitement, she cannot perform this office.

I recognize gold as superior to all other material in general that we have at our command, if skillfully manipulated. But it must be used with judgment, in its proper place. We must acknowledge its conductivity, consequently its inadaptability to meet the requirements of all cases.

The therapeutic effects of English precipitated chalk and lime water, would do much to stay the progress of decay in this class of teeth, by counteracting the acid condition of the secretion usually manifested in such cases. A hygienic diet with young subjects, would work a great change. But what can we effect in respect to hygiene? But little. People will eat what their appetites crave, if t is at the expense of teeth. The good advice that we may be inclined to give on this subject, will not be heeded by many. The topical treatment, conducted by the dentist, is about all that will effect much, aside from the beneficial results of ordinary cleaning. This, most all will do.

We must depend upon our own exertions; success is only accomplished by good judgment on our part, and a thorough knowledge of the requirements that each case demands.

The application of such remedies as carbolic acid, creasote, theymal, oxide of tin, and varnish, will effect much toward making perfect operations in gold a success. By the affinity of the tooth element, for remedies applied, the tubuli is sealed up in a measure and the aqueous element disposed of in a degree. Our object should be to prevent chemical action, by the application of such remedies as tend very much to dispose of the aqueous element. If this is accomplished, and gold operations are absolutely first-class, our efforts are generally crowned with success. If we do not succeed in controlling chemical action, non-conducting fillings will usually best meet the requirements. This precaution would often make a successful operation in gold that would otherwise prove a failure. In fact, the application of carbolic acid, or creasote, should never be omitted. For it disinfects the cavity, thereby acts as an antiseptic, obtends sensitiveness, lessens thermal shocks, and imparts general comfort and satisfaction to the patient.

When cavities are deep, it is usually best to line such with some non-conducting material, as a decided benefit will be derived from such a practice. It is plainly shown that a tooth affected with caries does not remain passive and inert to impressions of decay; but, on the contrary, great efforts are made by nature to protect pulps from exposure, and from carious influences. And the half decomposed calcarious condition of caries in cavities, or superficiallp located, is often transformed into a hard and bony-like consistency. A condition that further decay makes but slow progress in. Now this is the natural work of nature; it may be assisted, or retarded. Irritation will produce either.

A gutta-percha filling will be neutral, and allow nature to go on with her work uninterrupted in calcification of the fibrili.

An oxy-chloride filling will stimulate the pulp to an active condition, if rightly applied. If too strong, the hydro-chloride, an element that the material possesses, may inflame the pulp, and death of the organ may ensue.

An oxidized tin filling prevents galvanic action, an antiseptic condition is established, leaving the walls of the cavity in the most favorable state to resist decay.

Dr. S. B. Palmer tells us how amalgam fillings preserve the teeth. He says, "Amalgam at first acts in the same manner as gold—though in a less degree, because it is not so good a conductor. But later the poor amalgam becomes itself oxidized, and the current is then lessened. Also the metallic coloring matter thrown off by oxidation, is received into the softened bone, until the once positive element, becomes neutral."

Thus decay is held in check. A gold filling does not oxidize, and can not hold decay in check, like tin and amalgam, and as often manipulated and inserted, irritates the vital condition so that decomposition and disintegration is promoted. How so? The gold filling becomes, under some circumstances, an agent of circulation. It promotes chemical action, it excites or increases this by establishing a galvanic current, which acidifies saliva, and then aids in producing an agent like acid, which irritates the vital organization of the tooth, causing it to break down at the margin of the filling. Then we have a condition established, that we term electro-chemical decomposition. This same condition of things in "well calcified teeth" would not be likely to make any impression upon the organ in this direction.

Thus it is, that a gold filling, in certain locations, and in well calcified teeth, may last forty (40) years; where one, equally good, or better, in another class of teeth, might not last forty weeks.

The "new departure trio," Drs. Palmer, Flagg and Chase, have developed these principles. They have been recognized as existing by many, for years. They have demonstrated plainly to my mind, the cause and effect of this theory. In practical observation I have seen its working. It has better fitted me to accept the situation, and prepared me to battle with the enemy. It has shown me the remedy for this and that case. It has given me instructions how to master the elements of decay, upon scientific principles, instead of merely mechanical means.

These men are benefactors. They have put at the disposal of the profession those principles with which to hold in check this destructive element. It is not going to revolutionize operative dentistry, by causing men to discard gold, and stuff cavities with plastics. The "new departure" does not advise anything of the kind. It should not be assailed on the ground that it is degrading, and will lower the standard of dentistry. Gentlemen, it is the reverse. The proposition of my esteemed friend, Dr. Flagg, is: That you enlarge the field of usefulness and the work of conservation of the teeth.

He says: "Make a beginning with the tooth that you would ordinarily extract, and treat them. Make 'that' dentistry. Fill these with plastic fillings. It is from a desire that the forceps be laid aside that this advice is given." (Mark it.) "It is from a desire that our patients shall eat upon teeth, the roots of which are in their jaw. It is from a desire that they shall be exempt from the infliction of artificial work. It is from a desire to extend the blessings which the hand of our profession holds to bestow. It is that we may be sought, rather than avoided; that we may be extolled, rather than decried; that we may be esteemed, rather than censured; that rather than be feared, we may be loved and respected."—*Dental Advertiser.*

